# Jealousy as a Function of Rival Characteristics: Two Large Replication Studies and Meta-Analyses Support Gender Differences in Reactions to Rival Attractiveness But Not Dominance

**DOI:** 10.1177/0146167220904512

**Published:** 2020-03-10

**Authors:** Thomas V. Pollet, Tamsin K. Saxton

**Affiliations:** 1 Northumbria University, Newcastle upon Tyne, UK

**Keywords:** jealousy, rival characteristics, replication, evolutionary psychology, sex differences

## Abstract

Jealousy is a key emotion studied in the context of romantic relationships. One seminal study (Dijkstra, P., & Buunk, B. (1998). Jealousy as a function of rival characteristics: An evolutionary perspective. Personality and Social Psychology Bulletin, 24 (11), 1158–1166. https://doi.org/10.1177/01461672982411003) investigated the interactions between a participant’s gender and their reactions to the attractiveness or dominance of a romantic rival. In a vignette-based study, it was found that women’s jealousy was more responsive than men’s to a rival’s attractiveness, whereas in contrast, the rival’s dominance evoked more jealousy from men than from women. Here, we attempt to replicate these interactions in two samples (*N* = 339 and *N* = 456) and present subsequent meta-analyses (combined *N*s = 5,899 and 4,038, respectively). These meta-analyses showed a small, significant effect of gender on jealousy provoked by rival attractiveness, but no such response to rival dominance. We discuss the potential reasons for these findings and future directions for research on jealousy and rival characteristics.

The differences between men and women in the nature of their romantic jealousy have been studied in dozens of empirical research papers (reviews and meta-analyses in Buss, 2018; Carpenter, 2012; Edlund and Sagarin, 2017; Harris, 2003; Sagarin et al., 2012) and presented as a test case of predictions derived from evolutionary psychology (e.g., Sesardic, 2003). Men can be at risk of raising a child that they mistakenly believe to be a genetic relative as a consequence of their partner’s sexual infidelity. This is not a risk that women face, but in contrast, a woman’s reproductive success depends in part upon the resources brought by her partner, something that could be threatened by her partner falling in love with someone else (emotional infidelity), and channeling resources away. Given these differences in the threats faced by men and women, researchers have predicted and frequently found differences in how much men’s and women’s jealousy is provoked by sexual or emotional infidelity. In a typical research design, where people are asked to decide whether they would be more distressed by sexual or emotional infidelity, men tend toward the former more than women do, whereas the opposite pattern is true of women.

This research program is not without controversy (Buss, 2018; Carpenter, 2012; Edlund & Sagarin, 2017; Harris, 2003; Sagarin et al., 2012). Some researchers perceive that sex differences in jealousy exist because natural selection has acted directly and independently on men’s and women’s psychology to instill their specific natures, deriving from the differences in costs to men and women of a partner’s sexual or emotional infidelity (e.g., Buss, 2000). Others question the extent to which we need posit that differences between men and women have been so canalized by processes of natural selection. Harris’s (2003) socio-cognitive theory of jealousy does not throw aside the role of natural selection, but instead considers evolution to have shaped a cognition that can respond more flexibly to the environment. Under that formulation, jealousy might be provoked to the extent that people perceive that a rival challenges them in relation to their representations of themselves or threatens the rewards that they currently gain from a relationship. Alternatively again, other researchers have focused their attention on the biosocial constructions of differences between men and women in their behavior (Wood & Eagly, 2012).

Researchers who prefer more socially constructed explanations of gender differences in behavior have considered null findings or heterogeneity in findings of male/female differences in jealousy to be supportive of their theories, because they point out that the contingencies of social and cultural exposure will lead to variability across samples in terms of the differences between men and women. This position has fuelled ongoing debate over whether the noted differences in jealousy between men and women are only apparent in some research designs (see Carpenter, 2012; Edlund & Sagarin, 2017; Harris, 2003; Sagarin et al., 2012). Irrespective, there is greater consensus across the different camps that the documented gender differences in jealousy exist most clearly in people to the extent that they are young, or heterosexual, or students, or American (Carpenter, 2012; Harris, 2003; Sagarin et al., 2012).

## A Replication of Dijkstra and Buunk (1998)

Despite the raft of controversies, evolutionary thinking on jealousy has also been used to predict how men and women differ in terms of which traits of a potential rival should most provoke their jealousy, as in a seminal study by Dijkstra and Buunk (1998). Dijkstra and Buunk (1998) focused on differences between men and women in their reactions to the dominance and attractiveness of a potential rival. A man’s dominance might testify to his ability to provide resources (e.g., Buss, 1994), whereas a woman’s physical attractiveness might provide cues to her fertility, age, and physical condition (e.g., Symons, 1979). As such, these characteristics are associated with high-quality partners and desired differentially in men and women the world over (see Buss, 1989). Dijkstra and Buunk presented participants with vignettes that described imaginary interactions between a man and a woman, one of whom was the participant’s partner, and the other of whom was a rival. The authors hypothesized that women would be particularly jealous of female rivals who were attractive rather than unattractive, while dominance should not be of great importance. In contrast, men would be particularly jealous when the male rival was high rather than low in dominance, and attractiveness of the rival would matter less.

Dijkstra and Buunk (1998) conducted a three-way analysis of variance (ANOVA) and found a significant Gender × Attractiveness × Dominance interaction with a sample of 152 students. Yet, the key evidence presented by Dijkstra and Buunk were two further significant interaction tests in ANOVA (Gender × Attractiveness, Gender × Dominance). Participant gender interacted with the attractiveness of the rival, leading women to respond with more jealousy to an attractive rival, as opposed to an unattractive one, compared with men (interaction: $\eta_{p}^{2}=.033,$ based on our own calculations). In contrast, the dominance of the rival affected men to a greater degree than it did women (interaction: $\eta_{p}^{2}=.026,$ based on our own calculations). While the effects were statistically significant, their size was relatively small (Cohen, 1969).

Subsequent to Dijkstra and Buunk (1998), there has been a suite of papers examining rival characteristics and their effects on jealousy (e.g., Buunk and Dijkstra, 2001; Dijkstra and Buunk, 2002; O’Connor and Feinberg, 2012; Lei et al., 2019; Zurriaga et al., 2018; see the “Discussion” section for details). Beyond inspiring much other research, the study by Dijkstra and Buunk (1998) is also cited in handbooks on close relationships, evolutionary psychology, and social psychology (e.g., Brehm, 2002; Hendrick & Hendrick, 2000; Neuberg et al., 2010; Schmitt, 2005). Thus, it is important to reexamine this seminal study and conduct a close replication. The necessity of revisiting earlier findings is further underlined by the current replication crisis in psychology, generating momentum to reappraise earlier work (Open Science Collaboration, 2015). Independent replication is the cornerstone for psychological science (e.g., Zwaan et al., 2018).

We evaluate the same two key hypotheses as Dijkstra and Buunk (1998). We predict a two-way interaction between gender and attractiveness, with women surpassing men in terms of how much their jealousy is provoked by the attractiveness of the rival. We also predict a two-way interaction between gender and dominance of the rival, with men’s jealousy being more reactive than women’s to the rival’s dominance.

## Study 1

### Method

#### Participants

The sample size was determined by the time frame allocated to two Bachelor students who completed data collection. The target sample size was 2.5 times the sample of the original study (152 × 2.5 = *N* of 380), as recommended by Simonsohn (2015), of which we fell slightly short. Our target population was unmarried, young adults, who had experienced at least one romantic relationship (including ongoing relationships). Some participants completed the study online (*N* = 271), whereas others were approached on a campus of a large U.K. university (*N* = 98) and completed the study on a tablet or their own device. The restriction of being unmarried was added as married individuals might respond differently to questions about jealousy (White, 1981). Given that there were no statistically significant interactions between the study site (online vs. campus) and the manipulation (Attractiveness/Dominance) on jealousy, we merged the samples (*N* = 369). While Dijkstra and Buunk (1998) did not specify whether they applied this criterion, we limited the sample to self-identified heterosexual participants (*N* = 339; 225 women). The majority were current students (55%) and in a relationship (66%). The mean age was 22.48 years (*SD* = 3.75 years, range = 18–57 years); the age of the participants recruited by Dijkstra and Buunk (1998) is not reported, but they are described as undergraduates.

#### Materials

We attempted to follow the materials used by Dijkstra and Buunk (1998) in the original study, as closely as possible. The materials that we used are available on the Open Science Framework (OSF; https://osf.io/zytdx/).

##### Vignettes

Our vignettes presented the same scenario as Dijkstra and Buunk (1998). Participants read:

*You are at a party with your girlfriend [boyfriend], and you are talking with some of your friends. You notice your girlfriend [boyfriend] across the room talking to a man [woman] you do not know. You can see from his [her] face that he [she] is very interested in your girlfriend [boyfriend]. He [She] is listening closely to what she [he] is saying, and you notice that he [she] casually touches her [his] hand. You notice that he [she] is flirting with her [him]. After a minute, your girlfriend [boyfriend] also begins to act flirtatiously. You can tell from the way she [he] is looking at him [her] that she [he] likes him [her] a great deal. They are completely absorbed in each other.*

##### Dominance manipulation

Dijkstra and Buunk (1998) manipulated dominance perception via a vignette written to capture high- and low-dominance items of the Dominance subscale of a personality questionnaire (NPV; Luteijn et al., 1985). We replicated the text, but altered the Dutch forenames and the university name. The high-dominance description read as follows:

*You find out that your girlfriend is flirting with Jonathan, the man in this photo. Jonathan is a student at [Name of University where study was conducted] and is about the same age as you. Jonathan is also a teaching assistant and teaches courses to undergraduates. He is also president of a [Name of University where study was conducted] activities club that numbers about 600 members. Jonathan knows what he wants and is a good judge of character. Jonathan also often takes the initiative to do something new, and he has a lot of influence on other people. At parties, he always livens things up*.

The low-dominance version read as follows:

*You find out that your girlfriend is flirting with Jonathan, the man in the photo. Jonathan is a student at [Name of University where study was conducted] and is about the same age as you. Jonathan attends classes regularly and is one of the 600 members of an activities club at [Name of University where study was conducted]. Jonathan does not always know what he wants, and he often fails to understand what is going on in other people’s minds. Jonathan often waits for others to take the initiative and is rather compliant. At parties, he usually stays in the background.*

For (heterosexual) women, the name and gender of the partner and rival were altered (“Olivia” rather than “Jonathan”).

##### Attractiveness manipulation via photographs

We contacted Pieternel Dijkstra for access to the original photographs but these were unavailable given the time lag since the original study; the requirement for new photographs allowed us to select stimuli that exhibited contemporary hairstyles and image quality, and so we drew our stimuli from a database of standardized photographs (DeBruine & Jones, 2017) that had been prerated for attractiveness on a 7-point scale, from 1 = *not at all* to 7 = *very attractive*, as in Dijkstra and Buunk’s (1998) original study. We matched the attractiveness levels of the stimuli as closely as possible to the original study (attractive female: *M* = 4.2, “009_08.jpeg,” original study: *M* = 4.05; unattractive female: *M* = 1.6, “038_08.jpg,” original study: *M* = 1.05; attractive male: *M* = 4.4, “036_08.jpeg,” original study: *M* = 4.43; unattractive male: *M* = 1.5, “005_08.jpg,” original study: *M* = 1.05). All individuals were smiling in their picture and the stimuli were 350 × 350 pixels (72 dpi).

##### Ratings of jealousy and other feelings

After reading the vignette, participants used a 5-point scale (1 = *not at all* to 5 = *very‘X’*) to rate the extent to which the vignettes would lead them to feel: jealous, distrustful, suspicious, worried, betrayed, rejected, hurt, anxious, threatened, sad, and upset (whereby ‘X’ corresponds to the emotion). Following Dijkstra and Buunk (1998), we focus on the jealousy item.

##### Manipulation check

Participants completed a manipulation check on the attractiveness of the rival in the vignette by answering the following questions: “How attractive do you think the person in the photo is?” and “How attractive do you believe this person is, in comparison to yourself?” on a 7-point scale (1 = *very attractive*, 7 = *not very attractive* and 1 = *far more attractive*, 7 = *far less attractive*, respectively). To check the participants’ ratings of the rival’s dominance, participants were then asked to rate the rival on a 5-point scale to indicate how typical (1 = *not at all typical*, 5 = *very typical*) the following six characteristics were of the rival: assertive, self-confident, influential, good judge of character, extraverted, and socially competent.

##### Mate value

As in Dijkstra and Buunk (1998), we included six items on self-perceived mate value (e.g., “I can have as many sexual partners as I choose”) from Landolt et al. (1995). These formed a coherent scale (Cronbach’s α = .88, 95% confidence interval [CI] = [.85, .90]). Dijkstra and Buunk (1998) found that men and women differed in mate value, with women reporting greater mate value. Thus, they included this measure as a covariate in all their ANOVAs. It is unclear whether mate value is truly an extraneous variable, and so it is debatable whether it is necessary to account for it in the proposed ANOVAs (e.g., Jamieson, 2004; Schneider et al., 2015). In the “Results” section, we further discuss this issue.

##### Inclusion of Others in Self Scale (IoS)

Participants also completed the Inclusion of Others in Self Scale (IoS; Aron et al., 1992) to measure how close they believed themselves and their partner to be. They were asked to choose a response from seven Venn diagrams of overlap between themselves and their partner or previous partner based on how interdependent or independent they believed they were. This measure was not part of Dijkstra and Buunk’s (1998) paper, but was included for exploratory analysis for the Bachelor thesis projects which made use of our data; this variable is not analyzed here. This measure was completed after all the relevant measures for the replication study and therefore could not influence any outcomes of what we present below.

#### Procedure

The study and its protocol were approved by the University’s Ethics Committee. Participants were recruited via social media adverts or by direct approach by two undergraduates (one man, one woman) with a tablet on a university campus of a large U.K. university. Participants read an information sheet and then provided informed consent. Prior to reading the scenario, participants answered some questions on sociodemographics, their sexuality, and relationship status. Participants were then presented with the vignette which described their current partner (whether real or imagined) flirting with a member of the opposite sex. After reading this scenario, the participants were then randomly shown either the high- or low-dominance descriptor, accompanied by either the attractive or unattractive photograph (see above). Next, participants completed their ratings of jealousy and other feelings, then the manipulation check questions, then the mate value questionnaire, then the IoS (see above). Participants were then thanked and debriefed.

#### Data analysis

All analyses were conducted in R 3.6.1 (R Development Core Team, 2008). The analyses were preregistered following Brandt et al.’s (2014) replication recipe on the OSF. The data, code, and analysis document are all available from the OSF (https://osf.io/zytdx/).

### Results

#### Manipulation checks

##### Attractiveness

We replicated Dijkstra and Buunk’s (1998) findings that, among the male raters, an ANOVA that examined the impact of the two types of photographs (high vs. low rival attractiveness) and two types of vignettes (high vs. low rival dominance) on perceived rival attractiveness (“How attractive do you think the person in the photo is?”), provided evidence only for a significant main effect of attractiveness, *F*(1, 110) = 257.70, *p* < .0001, $\eta_{g}^{2}=.70.$ Men rated the attractive rival as more attractive (*M* = 3.04, *SD*= 1.28, original study: *M* = 2.59) than the unattractive rival (*M*= 6.24, *SD* = 0.78, original study: *M* = 4.92). The same ANOVA, but switching the dependent variable to “How attractive do you believe this person is, in comparison to yourself?,” again revealed a significant main effect of attractiveness, *F*(1, 110) = 38.91, *p* < .0001, $\eta_{g}^{2}=.26.$ Men gave higher ratings to the attractive (*M* = 3.86, *SD* = 1.48, original study: *M* = 2.82) than the unattractive rival (*M* = 5.66, *SD* = 1.64, original *M* = 5.61).

The manipulation checks similarly supported a successful manipulation of rival physical attractiveness among female participants. In the corresponding 2 × 2 ANOVAs, there was only a significant main effect of rival attractiveness, *F*(1, 221) = 259.54, *p* < .0001, $\eta_{g}^{2}=.54,$ on ratings of attractiveness, and *F*(1, 221) = 91.10, *p* < .0001, $\eta_{g}^{2}=.29,$ on ratings of attractiveness compared with the self. Women rated the attractive rival as more attractive (*M* = 2.80, *SD* = 1.65, original *M* = 2.40) than the unattractive rival (*M* = 5.41, *SD* = 1.27, original *M* = 5.09). Women’s ratings of rival attractiveness compared with themselves also were higher in relation to the attractive rival (*M* = 3.41, *SD* = 1.65, original *M* = 2.81) than to the unattractive rival (*M* = 5.36, *SD* = 1.34, original *M* = 5.61).

##### Dominance

Following Dijkstra and Buunk (1998), we conducted a 2 (high vs. low rival dominance) × 2 (high vs. low rival attractiveness) multivariate analysis of variance (MANOVA) on the six dominance traits. In line with the original study, participants who read the high-dominance version of the vignettes gave higher ratings to all six dominance traits (male participants: Pillai’s Trace= .51, *F*(6, 105) = 17.70, *p* < .0001; female participants: Pillai’s Trace= .24, *F*(6, 213) = 11.06, *p* < .0001). All of the *F* tests showed a statistically significant effect for dominance of the rival for each of the six traits (male participants: all *F*’s(1, 110) > 55, all *p*’s < .0001; female participants: all *F*’s(1, 218) > 22, all *p*’s < .0001). We did not find a statistically significant (*p* <.05) main effect of attractiveness on dominance ratings in men, Pillai’s Trace = .09, *F*(6, 105) = 1.64, *p* = .145 (compare Dijkstra and Buunk’s (1998) report of *p* = .05). For women, we did find a statistically significant main effect of attractiveness on dominance ratings, in line with Dijkstra and Buunk (1998), Pillai’s Trace = .16, *F* = 6.68, *p* < .0001. The *F* tests showed a statistically significant effect for attractiveness of the rival on assertiveness, self-confidence, extraversion, influence, and social competence, all *F*’s(1, 218) > 5, *p*’s < .05. The only exception was the trait of being a good judge of character, *F*(1, 218) = 3.70, *p* = .055. These results are largely similar to Dijkstra and Buunk (1998) who reported statistically significant effects for all traits apart from social competence and being a good judge of character.

In conclusion, our manipulations were successful and elicited largely similar effects as Dijkstra and Buunk (1998). As discussed by Dijkstra and Buunk (1998), one cannot expect a complete experimental disentanglement between the dominance and attractiveness manipulations, as, for example, a manipulation of attractiveness is predicted to also affect perceptions of overall character (Feingold, 1992).

#### Mate value

Dijkstra and Buunk (1998) added self-perceived mate value as a covariate in all of their ANOVAs. In our study, men’s self-perceived mate value, *M* = 24.53, *SD* = 7.06, did not differ significantly from women’s, *M* = 25.85, *SD* = 6.98; *t*(225.24) = 1.64, *p* = .102. We therefore do not include mate value as a covariate in the analyses presented below, although the results are qualitatively similar with the inclusion of the covariate (see analysis document on the OSF: https://osf.io/zytdx/).

#### Hypothesis tests

$$\begin{array}{l} 2\;\left(rival\;physical\;attractiveness\right)\times 2\;\left(rival\;dominance\right)\times 2\;\left(gender\right)\;\\ ANOVA:effects\;on\;jealousy\;ratings \end{array}$$

**Figure 1.**
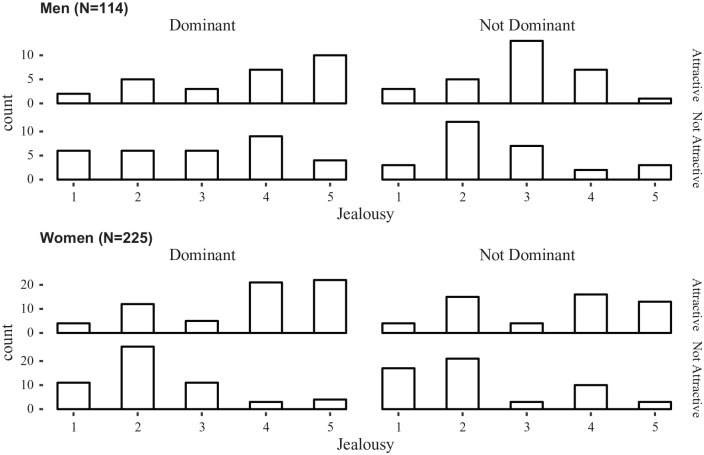
Histogram of number of male (top) and female (bottom) participants who gave each jealousy rating, separated by rival dominance (left and right set of graphs) and rival attractiveness (upper and lower graphs in each pair; Study 1).

Figure 1 presents the histograms by condition for men and women.

Unlike Dijkstra and Buunk (1998), the proposed 2 × 2 × 2 interaction (Gender × Attractiveness × Dominance) on ratings of jealousy was not statistically significant, *F*(1, 331) = 0.04, *p* = .849, $\eta_{g}^{2}<.01.$ Yet, there was evidence for the hypothesized Gender × Attractiveness interaction effect, *F*(1, 331) = 6.55, *p* = .011, $\eta_{g}^{2}=.02.$ For women, an attractive rival, as opposed to an unattractive rival, elicited jealousy to a greater degree than it did for men. There was no support for a Gender × Dominance interaction on jealousy, *F*(1, 331) = 1.44, *p* = .231, $\eta_{g}^{2}<.01.$ No other effects were statistically significant, including the main effect of dominance of the rival, *F*(1, 331) = 3.17, *p* = .076, $\eta_{g}^{2}<.01.$

##### Analyses of jealousy by gender

**Figure 2.**
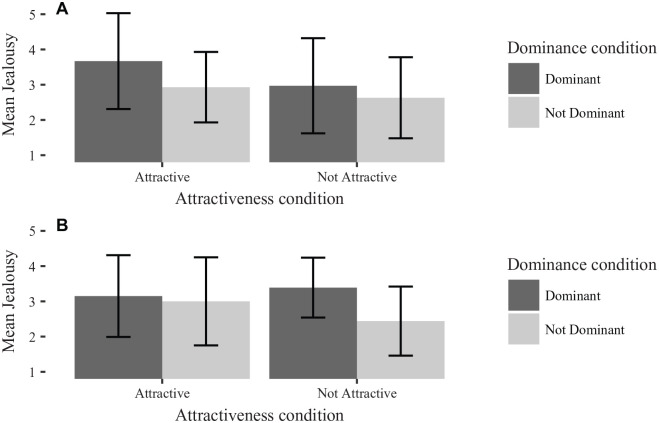
Bar chart of men’s jealousy separated by rival dominance and rival attractiveness, for (A) Study 1 and (B) Dijkstra and Buunk (1998). Error bars are *SD*.

**Figure 3.**
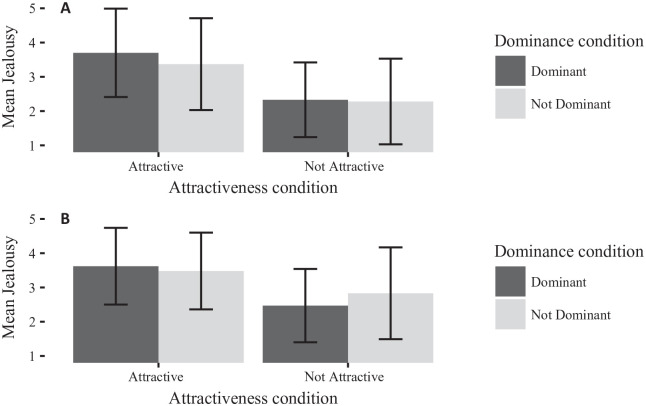
Bar chart of women’s jealousy separated by rival dominance and rival attractiveness, for (A) Study 1 and (B) Dijkstra and Buunk (1998). Error bars are *SD*.

Figures 2 and 3 show the effects of gender on ratings of jealousy, in comparison with the findings reported by Dijkstra and Buunk (1998). For men, a 2 (rival attractiveness) × 2 (rival dominance) ANOVA showed that men were significantly more jealous of attractive than unattractive rivals, *F*(1, 110) = 4.73, *p* = .032, $\eta_{g}^{2}=.04,$ and of high-dominance than low-dominance rivals, *F*(1, 110) = 5.45, *p* = .021, $\eta_{g}^{2}=.05.$ Unlike Dijkstra and Buunk (1998), there was no suggestion of an interaction effect, *F*(1, 110) = 0.75, *p* = .389, $\eta_{g}^{2}<.01.$ For women, a 2 (rival attractiveness) × 2 (rival dominance) ANOVA found only evidence for a main effect of attractiveness, *F*(1, 221) =54.43, *p* < .001, $\eta_{g}^{2}=.20.$ There was neither evidence for a significant effect of dominance of the rival, nor for the interaction effect between attractiveness and dominance; *F*(1, 221) = 1.34, *p* = .247, $\eta_{g}^{2}<.01$ and *F*(1, 221) = 0.75, *p* = .389, $\eta_{g}^{2}<.01,$ respectively.

### Discussion

The study that we attempted to replicate by Dijkstra and Buunk (1998) found that, in an imagined scenario when a participant watches their partner interact with a potential rival, women’s jealousy was provoked by the attractiveness of the female rival, whereas men’s jealousy was contingent upon the perceived dominance of the male rival. Specifically, the original paper found a significant three-way interaction between participant gender and the attractiveness and dominance of the rival; this was not something that we were able to replicate. The original paper also presented significant two-way interactions between participant gender and attractiveness, and between participant gender and dominance. We replicated the first but not the second of these two-way interactions: in our study, women’s jealousy was significantly more affected than men’s by the attractiveness of the rival. In analyses of men and women separately, we found that rival attractiveness but not rival dominance affected women’s jealousy ratings, whereas attractive or dominant rivals each increased men’s ratings of jealousy.

Sagarin et al. (2012) explain in detail why an interaction, and not main effects, is the only test of a hypothesis around evolved sex differences (see also Buller, 2005, on the importance of selecting the correct contrasts in investigating male/female differences in jealousy). It is true that the men in our study were more jealous of dominant than nondominant men, but they were also more jealous of attractive than unattractive men. We can infer that men are alert to socially desirable traits. The prediction of Dijkstra and Buunk, in contrast, states specifically that men, compared with women, should be more upset by dominance than attractiveness, because dominance is more threatening than attractiveness in the context of a male rival, and therefore we would predict interactions between gender and attractiveness, and gender and dominance (Dijkstra & Buunk, 1998, p. 1159).

It is not easy to explain the discrepancies between our findings and the findings of the original paper. Our manipulation checks demonstrated that our attractiveness and dominance manipulations affected the participants as intended, and our sample size was over twice that of the original. We do not have particular reason to believe that our participant sample differed sufficiently from the original to lead to the differences; Dijkstra and Buunk (1998) recruited undergraduates from a university in the Netherlands, whereas we focused our recruitment around a U.K. university (just over half of our participants were students), and we recruited participants with a mean age of 22 years. Dijkstra and Buunk (1998) state that the well-known Netherlandic culture of sexual equality makes that country a particularly rigorous test of male/female differences in jealousy, implying that men and women outside the Netherlands may be more likely to differ in the jealousy provoked by different rival characteristics. Although it is not necessarily borne out empirically that male/female differences are greater in non-egalitarian cultures (e.g., Buunk & Dijkstra, 2015), this statement does imply that we should not explain away our null findings based on that the data were collected outside the Netherlands. The original study took place two decades prior to our replication, and it is possible that a cultural shift or difference could explain the discrepant results; perhaps flirting is considered less consequential in our cohort, and so less likely to have serious ramifications. One other possible contributor to the failed replication is our stimuli photographs: the original photographs were not available, and so we used other stimuli that we matched approximately to the original in terms of rated attractiveness, but differed from the originals in other ways, including in particular ethnicity. We also fell short of our sample target. Accordingly, to try to verify our findings, we carried out a further replication.

## Study 2

### Participants

Participants were recruited from an online crowd-sourcing website (www.prolific.ac; Palan & Schitter, 2018). We aimed at a minimum sample 2.5 times the size of the original study (152 × 2.5 = *N* of 380), following Simonsohn (2015). The study was only advertised to potential participants who stated, when they enrolled with the crowd-sourcing website, that they were heterosexual students. Participants were paid £1 for their contribution to the study, leading to *N* = 404. This sample was supplemented with a small online sample who were recruited via social media and word of mouth (*N* = 52). We merged both samples for analyses (*N* = 456; 278 women). The majority were current students (81%) and in a relationship (61%). The mean age was 23.34 years (*SD* = 4.10 years, range = 18–56 years).

### Materials

The materials followed Study 1, with the minor exceptions described below. We no longer included the IoS.

#### Jealousy scenario

The scenario was the same as Study 1 and Dijkstra and Buunk (1998).

#### Dominance manipulation

The only deviation from Study 1 was that the vignette referred to “University” rather than the specific university named in Study 1.

#### Attractiveness manipulation via photos

We used photos from the Radboud Faces Database (Langner et al., 2010), which provides standardized photos prerated for attractiveness on a 5-point scale. We converted the ratings to a 7-point scale so that ratings were comparable with those used in the original study and selected faces so that the high- and low-attractiveness faces differed identically between the genders. The stimuli selected were all White and had a neutral expression (Rafd090_21_Caucasian_male_neutral_frontal.jpg, Rafd090_22_Caucasian_female_neutral_frontal.jpg, Rafd090_30_Caucasian_male_neutral_frontal.jpg, Rafd090_37_Caucasian_female_neutral_frontal.jpg). Crucially, the difference between the unattractive and attractive photos was identical (2.38 points on the 7-point scale) for men (mean ratings of 5.3 and 2.9) and women (mean ratings of 4.9 and 2.5). Further details can be found on the OSF (https://osf.io/wd7zv/).

#### Mate value

The six items formed a highly reliable scale (Cronbach’s α = .91; 95% CI = [.89, .92]).

### Procedure

The procedure was the same as Study 1, with the exception that we no longer included the IoS, and that different populations were recruited.

### Results

#### Manipulation checks

##### Attractiveness

Replicating Dijkstra and Buunk (1998), a 2 × 2 ANOVA (high vs. low rival attractiveness; high vs. low rival dominance) on men’s ratings revealed a significant main effect of manipulated rival attractiveness on perceived rival attractiveness, *F*(1, 174) = 88.88, *p* < .0001, $\eta_{g}^{2}=.34.$ Men rated the attractive rival as more attractive (*M* = 4.06, *SD*= 1.18, original *M* = 2.59) than the unattractive rival (*M*= 5.52, *SD* = 0.96, original *M* = 4.92). Dominant rivals were also perceived as more attractive, *F*(1, 174) = 15.86, *p* < .001, $\eta_{g}^{2}=.08,$ although this effect was more than 4 times smaller than the effect of attractiveness on perceived attractiveness. The interaction was not statistically significant, *F*(1, 174) = 0.36, *p* = .547, $\eta_{g}^{2}<.01.$ Similarly, we found that in the male sample, a 2 (rival physical attractiveness) × 2 (rival dominance) ANOVA on perceived rival attractiveness compared with oneself supported a significant main effect of the attractiveness manipulation, *F*(1, 174) = 31.99, *p* < .001, $\eta_{g}^{2}=.16,$ and a main effect of the dominance manipulation, *F*(1, 174) = 7.64, *p* = .006, $\eta_{g}^{2}=.04.$ The interaction effect was not statistically significant, *F*(1, 174) = 0.89, *p* = .348, $\eta_{g}^{2}<.01.$ Again, the effect was roughly fourfold for the attractiveness manipulation as opposed to the dominance manipulation. Thus, we can conclude that the manipulation was successful: compared with themselves, men rated the attractive rival as more attractive (*M* = 4.23, *SD* = 1.41, original study: *M* = 2.82) than the unattractive rival (*M* = 5.40, *SD* = 1.41, original study: *M* = 5.61).

The manipulation checks similarly supported a successful manipulation of rival physical attractiveness on women’s ratings. In the two corresponding 2 × 2 ANOVAs, there was a statistically significant main effect of photograph attractiveness, *F*(1, 274) = 67.13, *p* < .0001, $\eta_{g}^{2}=.20,$ and *F*(1, 274) = 26.43, *p* < .0001, $\eta_{g}^{2}=.09,$ respectively. Women rated the attractive rival as more attractive (*M* = 3.62, *SD* = 1.38, original *M* = 2.40) than the unattractive rival (*M* = 4.94, *SD* = 1.30, original *M* = 5.09). In comparison with themselves, women also rated the attractive rival as more attractive (*M* = 4.22, *SD* = 1.50, original *M* = 2.81) than the unattractive rival (*M* = 5.12, *SD* = 1.45, original *M* = 5.61). In the 2 × 2 ANOVA on attractiveness in comparison with oneself, there was also a significant interaction between rival attractiveness and rival dominance, *F*(1, 274) = 6.29, *p* = .013, $\eta_{g}^{2}=.02,$ but this effect was roughly a quarter of the size of the main effect of attractiveness. Taken together, this suggests that we successfully manipulated rival attractiveness for the female participants.

##### Dominance

For men, a 2 × 2 MANOVA showed a significant effect of the dominance manipulation on ratings of the six rival dominance traits, Pillai’s Trace= .28, *F*(6, 169) = 11.07, *p* < .0001. All of the univariate *F* tests showed a statistically significant effect for dominance of the rival, all *F*’s(1, 174) > 19, all *p*’s < .0001. Similarly, for women, the 2 × 2 MANOVA supported the successful manipulation of dominance for all six ratings, Pillai’s Trace= .40, *F*(6, 269) = 29.53, *p* < .0001. All of the univariate *F* tests showed a statistically significant effect for dominance of the rival, all *F*’s(1, 276) > 49, all *p*’s < .0001.

The 2 × 2 MANOVA in men also showed a significant effect of rival attractiveness on ratings of the six dominance traits, Pillai’s Trace= .07, *F*(6, 169) = 11.07, *p* = .035. This is similar to the result reported by Dijkstra and Buunk, *F*(6, 65) = 2.33, *p*=.05. Note that the effect of dominance is 4 times the size of that of attractiveness (Pillai’s Trace = .28 vs. Pillai’s Trace = .07). The follow-up ANOVAs showed a statistically significant effect of attractiveness on ratings of assertiveness, self-confidence, extraversion, social competence, all *F*’s(1, 174) > 4.5, all *p*’s < .05, but no statistically significant effect on ratings of “being a good judge of character,” *F*(1, 174) = 0.18, *p* = .668, and on ratings of influence, *F*(1, 174) = 3.32, *p* = .070. Unlike men, and unlike Study 1 and Dijkstra and Buunk (1998), we found no significant effect of rival attractiveness on ratings of the six dominance traits in the 2 × 2 MANOVA, Pillai’s Trace = .03, *F*(6, 269) = .07, *p* = .167.

##### Mate value

Unlike Study 1, women (*M* = 24.39, *SD* = 7.99) compared to men (*M* = 22.63, *SD* = 7.6) reported a significantly higher self-reported mate value, *t*(391.19) = 2.36, *p* = .019, but this was a small effect, Cohen’s *d* = 0.22, 95% CI = [0.03, 0.41]. Inclusion of a covariate could lead to issues (Schneider et al., 2015), and given that the effect was small, and to maintain consistency with Study 1, we did not include the covariate in our ANOVA design. Including mate value as a covariate in the 2 × 2 × 2 ANOVA leads to similar conclusions as those described below (none of the effects were statistically significant, all *p*’s > .19, analyses described in full in the analysis document on the OSF).

#### Hypothesis tests

$$\begin{array}{l} 2\left(rival\;physical\;attractiveness\right)\times 2\;\left(rival\;dominance\right)\times 2\;\left(gender\right)\\ ANOVA:effects\;on\;jealousy\;ratings \end{array}$$

**Figure 4.**
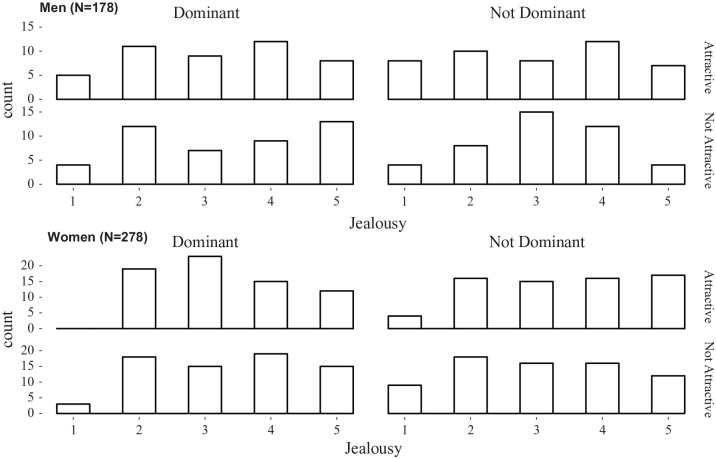
Histogram of number of male (top) and female (bottom) participants who gave each jealousy rating, separated by rival dominance (left and right set of graphs) and rival attractiveness (upper and lower graphs in each pair; Study 2).

Figure 4 presents the histograms by condition for men and women.

In line with Study 1, but unlike Dijkstra and Buunk (1998), the proposed 2 × 2 × 2 interaction was not statistically significant, *F*(1, 448) = 0.42, *p* = .518, $\eta_{g}^{2}<.01.$ Contrary to both Study 1 and Dijkstra and Buunk (1998), there was no statistical evidence for the hypothesized Gender × Attractiveness interaction on jealousy, *F*(1, 448) = 1.23, *p* = .268, $\eta_{g}^{2}<.01.$ There was also no support for a Gender × Dominance interaction, *F*(1, 448) = 0.15, *p* = .694, $\eta_{g}^{2}<.01$. No other effects were statistically significant (all *p*’s >. 29).

##### Analyses of jealousy by gender

**Figure 5.**
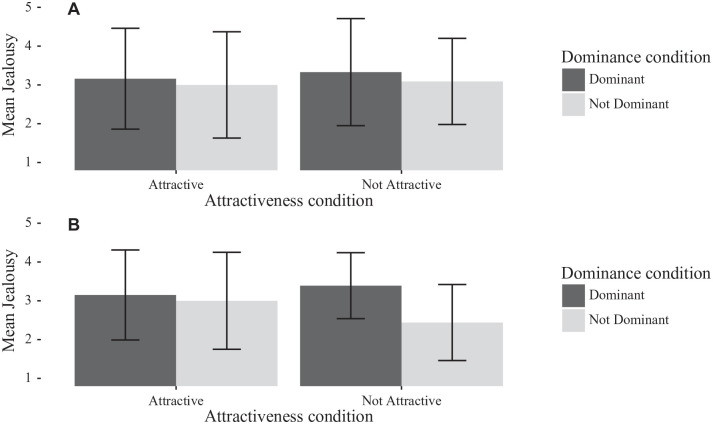
Bar chart of men’s jealousy separated by rival dominance and rival attractiveness, for (A) Study 2 and (B) Dijkstra and Buunk (1998). Error bars are *SD*.

**Figure 6.**
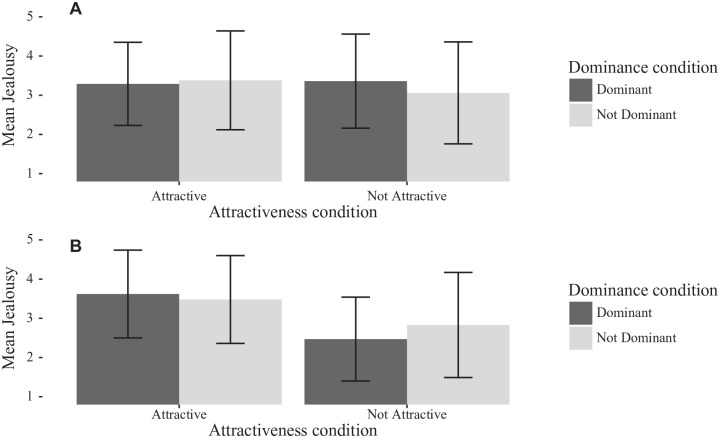
Bar chart of women’s jealousy separated by rival dominance and rival attractiveness, for (A) Study 1 and (B) Dijkstra and Buunk (1998). Error bars are *SD*.

Figures 5 and 6 show the effects by gender in comparison with the original study. For men, a 2 (rival physical attractiveness) × 2 (rival dominance) ANOVA showed no significant main effects of attractiveness or dominance on jealousy, nor an interaction (all *F*’s < 1.1, all *p*’s > .3). Similarly, for women, a 2 (rival physical attractiveness) × 2 (rival dominance) ANOVA showed no significant effects (all *F*’s < 1.85, all *p*’s > .17).

### Discussion

None of the analyses supported the hypothesized interaction effects. Given that we were left with mixed findings, we conducted a meta-analysis of Study 1, Study 2, and all of the relevant published findings that we could locate, to provide synthesis. Our meta-analysis additionally allowed us to include leave-one-out analyses (see supplementary materials on the OSF, https://osf.io/wd7zv/) to confirm that results were robust to the exclusion of individual studies.

### Meta-Analytic Synthesis

**Figure 7.**
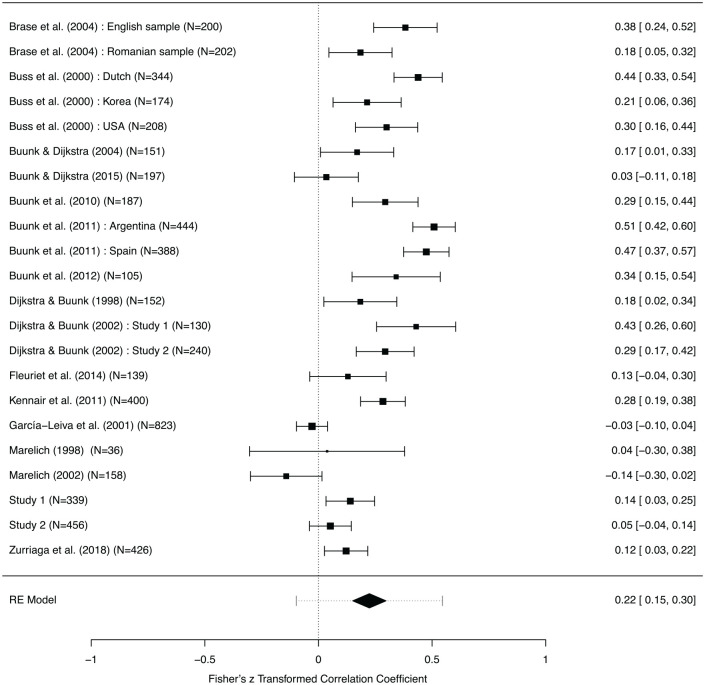
Forest plot (effects and 95% Confidence Interval) for gender differences in the effect of rival attractiveness on jealousy. *Note.* Note that the horizontal dashed interval for the Random Effects model (“RE Model”) is the prediction interval.

We searched Web of Science and located 198 papers that used the term “jealousy,” plus either “partner” or “rival,” plus either “trait” or “characteristic” or “attribute” or “quality” or “feature” (and variants of those words such as “traits”). We also obtained 27 candidate papers via Google Scholar (as they cited Dijkstra & Buunk, 1998, or similar papers). After excluding duplicates and screening, 16 of these papers were deemed relevant (description of criteria at https://osf.io/wd7zv/), and 15 yielded usable effect sizes representing gender differences in reaction to attractiveness or dominance of a rival (no effect size derivation possible for Nadler & Dotan, 1992). Of the 22 samples that we used (see Figure 7), five specified that participants were exclusively heterosexual and none focused exclusively on nonheterosexual participants; 17 used samples whose mean age was <26 years, three used samples whose mean age was > 26, and two did not provide participant ages; 15 used student (or majority student) participants and six did not (one unspecified); four samples were collected within the United States, whereas the remainder were based outside the United States.

We converted the usable effect sizes to Pearson correlations, and then applied Fisher’s *r* to *z* transformation. We then conducted random effects meta-analyses with REML estimation via the metafor package in R to examine how men’s and women’s jealousy was affected by rival attractiveness and rival dominance (Viechtbauer, 2010, 2015). All details, including additional tests and checks (e.g., funnel plots and leave-one-out analyses), can be found on the OSF (https://osf.io/wd7zv/).

Meta-analysis supported a weak effect for a gender difference in how rival attractiveness affected jealousy (*k* = 22 samples encompassing 5,899 participants, *r* = 0.22, 95% CI = [0.15, 0.3]; Figure 7). A visual check suggested no evidence of publication bias. There was, however, substantial heterogeneity in the effect, *Q*(21) = 194.83, *p* < .0001, $I^{2}=86.91\%,$ $\tau^{2}=.026.$

**Figure 8.**
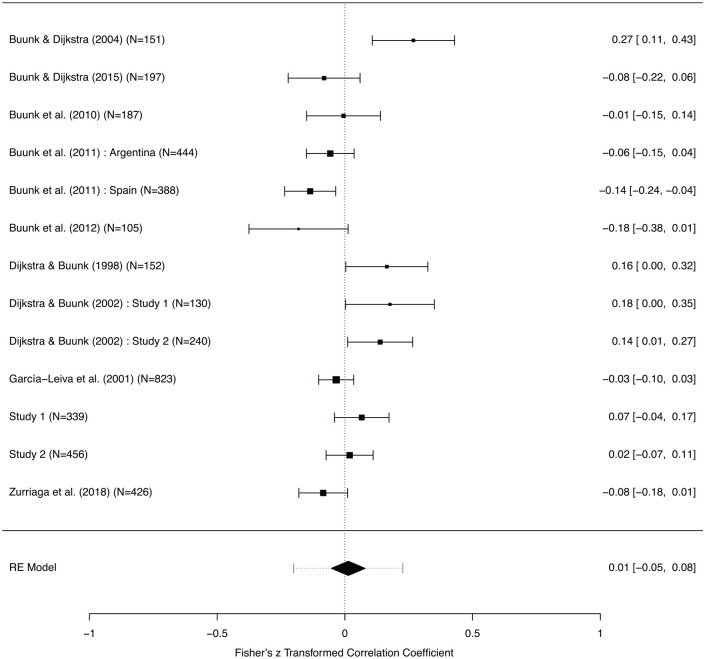
Forest plot (effects and 95% Confidence Interval) for gender differences in the effect of rival dominance on jealousy. *Note.* Note that the horizontal dashed interval for the Random Effects model (“RE Model”) is the prediction interval.

In contrast, although notably based upon a smaller sample (*k* = 13 samples encompassing 4,038 participants), there was no support for a gender difference in how social dominance of the rival affected reported jealousy (*r* = 0.01, 95% CI = [−0.05, 0.08]; Figure 8). Again, a visual check suggested no evidence of publication bias. There was substantial heterogeneity in the effect, *Q*(12) = 41.77, *p* < .0001, $I^{2}=76.15\%,$ $\tau^{2}=.011.$

A reviewer suggested that we conduct meta-regression to further examine the effect of several potential moderators on the effect (e.g., age of participants, study design). Meta-regression is especially likely to yield false-positive results when the number of studies is low, when there is a large number of potential moderators, and when heterogeneity is present (Higgins & Thompson, 2004). In the absence of strong a priori predictions, we therefore did not pursue meta-regressions. This is in line with recommended best practice (e.g., Higgins & Thompson, 2004). Nonetheless, in the “General Discussion” section, we suggest some candidate moderators, but we believe that these should be explored in line with theoretical motivations, and with a larger number of studies, in a structured, preregistered way, to avoid overfitting.

We therefore conclude that, all together, there is a small, significant effect of gender on jealousy provoked by rival attractiveness, such that rival attractiveness influences women’s reports of jealousy to a greater degree than it influences men’s reports. There is no good evidence for a robust gender difference in jealousy responses to rival dominance.

## General Discussion

We set out to perform a direct replication of a well-cited study, Dijkstra and Buunk (1998), that found that in a vignette-based scenario where participants imagined their partner being approached by a potential other-sex romantic rival, the men’s jealousy appeared to be particularly responsive to the dominance of the male rival, whereas the women’s jealousy appeared to be particularly responsive to the attractiveness of the female rival. This male/female difference was predicted based on evolutionary theory regarding the relative importance of dominance and attractiveness to men’s and women’s appeal as a romantic partner. In two empirical studies plus a meta-analysis that drew from an additional 15 published papers sampling nearly 6,000 participants, we found evidence that the attractiveness of a rival provoked women’s jealousy, and did so to a greater extent than it did men’s, but the overall effect size was small, and the published findings demonstrated substantial heterogeneity. The subset of the papers (13 samples; more than 4,000 participants) that focused on a rival’s dominance provided no good evidence that this affected men’s jealousy to a greater extent than it does women’s; again, findings across the literature were heterogeneous, as is typical for psychology (e.g., Kenny & Judd, 2019). The heterogeneity in effect sizes implies first that we should treat estimates of the average effect size with caution, and second that we might better understand the phenomenon under investigation if we explore the sociocultural or methodological influences that contribute to the variability in the size of the difference between men and women.

There are two principal design limitations that might help explain why studies in this area do not consistently find gender differences in jealousy. The first is the use of vignettes, which allow researchers to simulate the topic of interest, but of course lack the depth and immersion of real life (Hughes & Huby, 2002). The vignette’s description, of the apparent rapid escalation of a nascent romantic attraction between a stranger and someone in a relationship, or the realization of a potentially ongoing infidelity, may not feel realistic for many participants. A textual manipulation might additionally lack realism for contemporary samples who would be more used to today’s regular exposure to interactive media. If this is the case, the vignettes might have been ineffective in provoking jealousy in some samples, and thus inadequate to robustly provoke different levels of jealousy between men and women, leading to null findings. The second limitation is the use of simple pseudoreplication in stimuli, a problematic design whereby hypotheses about a class of stimuli are tested using just one (or a few) exemplar(s) (e.g., Hurlbert, 1984; Kroodsma et al., 2001; Wells & Windschitl, 1999). Thus, following the design of the study that we sought to replicate, our study design used just one stimulus to represent each of the high-dominance and low-dominance rivals, and just one male and one female photograph to represent each of the attractive and less attractive rivals. Even given our successful manipulation checks, the stimuli could have been inadequate as a solid representation of their class of stimuli. As a specific example of how this could be problematic, attractiveness is associated with a whole range of different parameters (e.g., symmetry, averageness, and apparent health; Rhodes, 2006) which would be represented to different degrees in the different stimuli used, and it is conceivable that differences in these parameters could mean that the different stimuli used in different studies agitate jealousy to greater or lesser extents, even if they are sufficient to pass the manipulation checks. Furthermore, it is possible that the hypothesized effects were not readily apparent in our replication studies because our participants were insufficiently motivated or engaged. However, our participants were drawn from standard sources of psychological data. Our participants, unlike those of the original study, were predominantly sourced online. Although early critiques of online studies expressed concerns about lack of quality control over the data, several studies have indicated that we do not need to have prima facie concerns that online studies are less reliable than offline studies (Krantz et al., 1997), and indeed online studies benefit from being able to reach large sample sizes (Birnbaum, 2004; Epstein et al., 2001; Krantz & Dalal, 2000), which can offset any increased noise in the data.

A productive direction for future research might be to consider the boundaries of any effect: Do rival characteristics shape jealousy in friendships, or sibling rivalries, for instance? The conventional study design on heterosexual male/female differences in responses to rival characteristics presents a perfect confound between rater and rival gender: Men judge male rivals, whereas women judge female rivals. This design does not allow us to rule out the possibility that the presumed domain-specific responses to rivals arise because men and women place different emphasis on dominance and attractiveness in judging others in all or many contexts. Indeed, differences in men’s and women’s use of the scales, or understanding of the concepts of attractiveness and dominance, could also add noise to the data (see Edlund and Sagarin, 2009, for discussion). Future research might also look beyond WEIRD populations (Western, Educated, Industrialized, Rich, Democratic; Henrich et al., 2010; Pollet & Saxton, 2019). We made use of a WEIRD sample, which was important to ensure compatibility with the original paper, but we should not assume the cross-cultural invariance of our findings. We believe that our results would be reproducible within other cohorts of young heterosexual adults in Western populations, who have at least some experience of romantic relationships. The appropriateness of the stimuli for the participants is also likely to be a key predictor of the success of the manipulation: for instance, whether the scenario in the vignette seems realistic to participants, and whether the images used to manipulate attractiveness of the rivals are suitable (e.g., in terms of age). We might expect different patterns of responses to rival characteristics in homosexual participants (Buunk & Dijkstra, 2001), or when people are focused on exclusively sexual infidelity without elements of emotional infidelity (Buunk & Dijkstra, 2004).

What do our results have to say about the impact of rival characteristics in jealousy? We do not doubt that individuals could be more or less intimidating as rivals, contingent upon their characteristics, including, in many circumstances, their dominance and attractiveness. However, our findings indicate that dominance, and even to some extent attractiveness, are not rival characteristics that distinguish men’s and women’s jealousy both reliably and substantially. This is perhaps not surprising, taken in the round. First, adults with established romantic relationships might adjust their jealousy based more upon their perceptions of the stability of their relationship, and the nature of their partner, than upon the characteristics of an abstract rival. They might also have a more precise idea of exactly which characteristics are considered particularly beguiling by their partner, and whether those characteristics are represented by the stimuli used or not. Second, the original study argues for women’s attraction to dominance on the basis that dominance relates to resource provision (Dijkstra & Buunk, 1998). While resource provision has been robustly demonstrated to be especially appealing to women (e.g., Buss, 1989), dominance (or, indeed, the set of traits manipulated by the vignette) is one step removed. Finally, there are also relevant individual differences that will interact with the stimuli, including the features that people find physically attractive (e.g., Lee et al., 2014), and the extent to which women seek dominance (or related constructs) in a partner (e.g., Lukaszewski & Roney, 2009). Overall, we conclude that the attractiveness and dominance of potential rivals are certainly characteristics that can be weighted in judging a rival’s threat, but the threat potential of those characteristics depends upon much more than gender.

## Supplemental Material

Pollet_Online_Appendix – Supplemental material for Jealousy as a Function of Rival Characteristics: Two Large Replication Studies and Meta-Analyses Support Gender Differences in Reactions to Rival Attractiveness But Not Dominance Click here for additional data file. Supplemental material, Pollet_Online_Appendix for Jealousy as a Function of Rival Characteristics: Two Large Replication Studies and Meta-Analyses Support Gender Differences in Reactions to Rival Attractiveness But Not Dominance by Thomas V. Pollet and Tamsin K. Saxton in Personality and Social Psychology Bulletin
