# Supplementary material for: Jealousy as a Function of Rival Characteristics: Two Large Replication Studies and Meta-Analyses Support Gender Differences in Reactions to Rival Attractiveness But Not Dominance
Source: Pers Soc Psychol Bull. 2020 Mar 10;46(10):1428–43. doi: 10.1177/0146167220904512 (PMC7493204; doi:10.1177/0146167220904512)
Supplement: Pollet_Online_Appendix – Supplemental material for Jealousy as a Function of Rival Characteristics: Two Large Replication Studies and Meta-Analyses Support Gender Differences in Reactions to Rival Attractiveness But Not Dominance [file Pollet_Online_Appendix.pdf]

All the materials, data, and code underlying our paper can be found online at the OSF project pages:
https://osf.io/zytdx/?view_only=e48db3ddebde41528741d04e814f44ff and https://osf.io/wd7zv/?view_only=6cd0b8ac87344a10a785a693b4041c12. They are not reproduced here due to length. All key materials are also summarised in text.
